# The Immune Response Against Human Cytomegalovirus Links Cellular to Systemic Senescence

**DOI:** 10.3390/cells9030766

**Published:** 2020-03-20

**Authors:** John J. Heath, Michael D. Grant

**Affiliations:** 1 Immunology and Infectious Diseases Program, Division of BioMedical Sciences, Faculty of Medicine, Memorial University of Newfoundland, 300 Prince Philip Drive, St. John’s, NL A1B 3V6, Canada; johnheath346@gmail.com; 2 Lady Davis Institute for Medical Research, Jewish General Hospital, Division of Experimental Medicine, McGill University, Montreal, QC H3A 0G4, Canada

**Keywords:** cytomegalovirus, clonal selection, telomere, senescence, inflammaging

## Abstract

Aging reflects long-term decline in physiological function and integrity. Changes arise at a variable pace governed by time-dependent and -independent mechanisms that are themselves complex, interdependent and variable. Molecular decay produces inferior cells that eventually dominate over healthy counterparts in tissues they comprise. In a form of biological entropy, progression from molecular through cellular to tissue level degeneration culminates in organ disease or dysfunction, affecting systemic health. To better understand time-independent contributors and their potential modulation, common biophysical bases for key molecular and cellular changes underlying age-related physiological deterioration must be delineated. This review addresses the potential contribution of cytomegalovirus (CMV)-driven T cell proliferation to cellular senescence and immunosenescence. We first describe molecular processes imposing cell cycle arrest, the foundation of cellular senescence, then focus on the unique distribution, phenotype and function of CMV-specific CD8^+^ T cells in the context of cellular senescence and “inflammaging”. Their features position CMV infection as a pathogenic accelerant of immune cell proliferation underlying immune senescence. In human immunodeficiency virus (HIV) infection, where increased inflammation and exaggerated anti-CMV immune responses accelerate immune senescence, CMV infection has emerged as a major factor in unhealthy aging. Thus, we speculate on mechanistic links between CMV-specific CD8^+^ T-cell expansion, immune senescence and prevalence of age-related disorders in HIV infection.

## 1. Cellular Senescence

To senesce, from the Latin *senescere,* is defined as “to grow old”. In biological terms, the concept of senescing or becoming senescent originates from work by Hayflick et al., describing loss of replicative capacity or onset of replicative senescence in fibroblast cell cultures [1,2]. While these observations addressed only one extrinsic aspect of senescence (inability to proliferate), they fueled speculation on an underlying mechanism, dependent on time in culture, that ultimately limits somatic cell replication. In this context, cell cycle arrest is perceived as a cellular anti-cancer response initiated following detection of aberrations in nuclear or mitochondrial DNA. Transient cell cycle arrest allows for evaluation of the internal damage, potential repair and eventual return to cell cycle progression. If internal cues for cell cycle arrest convey high risk for neoplastic transformation or oncogenesis, the cell proceeds through apoptosis and is eliminated or maintains cell cycle arrest, becoming senescent. Although classically defined by permanent cell cycle arrest and apoptosis resistance, later studies of cellular senescence revealed an operational range well beyond tumor suppression [3]. In fact, cellular senescence is now viewed as a dynamic process, integral to cellular and tissue aging and, conversely, to tissue homeostasis. Senescent differentiation spans all cell types and tissue microenvironments, each with its own associated functional alterations and contribution to organismal aging. Unravelling the complexity and pleiotropy of senescent processes will elucidate not only endogenous mechanisms of aging and key environmental stressors, but also homeostatic mechanisms integral to cancer prevention and wound healing.

**Figure 1:**
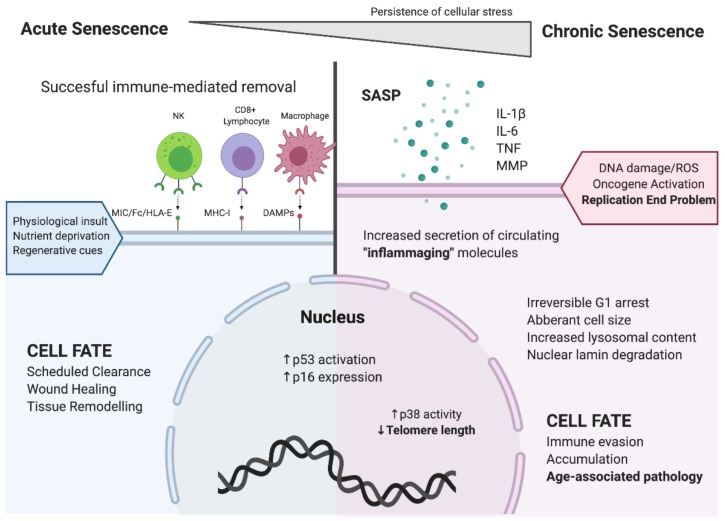
Distinguishing features of acute and chronic cellular senescence. Acute cellular senescence, depicted on the left side of Figure 1, involves coordinated recognition of senescent cells by immune cells, leading to their rapid elimination and replacement by healthy cells. Chronic cellular senescence, depicted on the right side of Figure 1, involves the accumulation of dysfunctional senescent cells, with local and systemic manifestation of the senescence associated secretory phenotype (SASP). Chronic inflammation linked to the accumulation of senescent cells with the SASP promotes further cellular senescence and age-associated pathology. (Created with BioRender).

Cellular senescence can be viewed as a programmed response to stressful events imposed through acute or chronic pressures. Much like the immune response to acute viral infection, acute cellular senescence is rapid and well orchestrated, ultimately shifting to senescent cell clearance and achieving a new equilibrium, as illustrated in Figure 1. Chronic senescence, like the immune response in chronic infection, is a progressively degenerative state linked to age-related pathology [4]. Acute cellular senescence is essential for tissue repair [5] and critical to physiological processes such as embryogenesis and maternal vascular remodeling [6,7,8]. Its key features are rapid onset and subsequent immune clearance of senescent cells. Acutely senescent cells receive short high-grade extracellular damage signals, which trigger the intracellular accumulation of stress signaling molecules that impose cell cycle arrest. Acute cellular senescence also rapidly invokes a distinct phenotypic change enabling immune selective elimination of senescent cells, thus, terminating with senescent cell clearance to maintain or restore proper tissue function (Figure 1) [4]. Degeneration of well-orchestrated senescent cell clearance and the subsequent accumulation of senescent cells underlies the canonical connection between chronic cell senescence, tissue deterioration and unhealthy aging.

As first demonstrated by Hayflick et al., long-term passage of fibroblast cell lines in culture terminates in permanent cell cycle arrest indicating chronic cellular senescence [1]. To link loss of replicative capacity in vitro to an in vivo process, Harley et al. later demonstrated that cultured primary human fibroblasts undergo declines in replication capacity over time periods directly proportional to the age of the donor and critically, that this decline is physically reflected in decreasing telomere length [9]. Chronic cellular senescence occurs via successive DNA damage responses induced by recognition of precariously shortened telomeres [10]. Telomere DNA sequences are noncoding and essentially serve as a protective cap to the extremities of linear chromosomes. When damaged or incompletely replicated, cell death may be triggered to avert chromosomal susceptibility to harmful mutations. Telomeres are comprised of hexameric TTAGGG repeats with overall length ranging from ~18 kilobase (kb) pairs in human cellular infancy to several kb pairs in old cells [11,12]. This in vivo connection to aging places telomere length as a cellular measure of age, or intrinsic ‘biological clock.’ The physical location of telomeres is both strategic to stabilize chromosomal DNA and the “Achilles heel” of long-term chromosomal stability as telomeres undergo successive loss with each cell division [9]. Chromosomal coding DNA remains protected during cell division at the expense of telomere length as follows. Most DNA polymerases utilized during cell division operate in a unidirectional 5′–3′ direction and can only bind to existing primer regions, creating problems for the Okazaki fragments of the lagging strands (3′-5′) where RNA primers are required. Since there is no site for attachment preceding the most 5′ RNA primer, when RNA primers are degraded post-DNA replication, a 3′ overhang of approximately 25 base pairs remains exposed and is eventually degraded. Thus, through this end-replication problem, telomere erosion occurs with every cell division [13]. An RNA-dependent DNA polymerase called telomerase can restore sections of the TTAGGG sequence during DNA replication and effectively eliminate the end replication problem. While this telomere maintenance strategy is employed by embryonic and other stem-like cells [14], telomerase is generally absent in differentiated cells in vivo [15] and telomere erosion is an unavoidable consequence of cell division. Lymphocytes are unique among differentiated cells in their capacity to upregulate telomerase upon activation, but this capacity is gradually lost upon repeated cycles of activation [16,17]. Therefore, telomere length reflects both the history of and, potential for further cellular replication in somatic tissue.

Due to this end replication problem, telomeres of cells undergoing extensive proliferation can ultimately shorten to dangerous lengths, leaving chromosomes ‘uncapped.’ These exposed ends mimic double-strand DNA breaks (DSBs) and trigger DNA damage response (DDR) pathways involving the master regulator protein p53 [18]. Senescence is initiated through p53-dependent expression of cyclin-dependent kinase inhibitors p16 and p21 that impose irreversible cell cycle arrest. Locked in the G_0_/G_1_ phase, downstream signaling cues initiated by p16 and p21 institute chromatin remodeling and genomic structural alterations that affect gene accessibility and expression [19,20,21]. Therefore, cellular processes become aberrant or dysfunctional and the cell develops a unique senescence-based phenotype reflecting its original cell type and previous associated functions, but functionally compromised. Identifying how senescent cell accumulation occurs in specific and general tissue microenvironments in vivo and how this impacts physiology is critical for understanding the physiology and pathophysiology of aging.

Processes such as telomere erosion and other progressive endogenous microaggressions persistently stress cells. Chronic, cumulative stress on cellular homeostasis does not overwhelm cells into acute senescence, but eventually culminates in chronic senescence. Chronic exposure to endogenous triggers is experimentally mimicked using low dose irradiation to create genotoxic DSBs and chromosomal aberrations that induce a senescent state similar to that reported by Hayflick in vitro. Irradiation of young healthy mice increases tissue expression of the cell cycle arrest marker, p16. Cells exhibiting markers of damaged chromatin, such as the phosphorylated histone variant H2AX, arise in tissue areas with extensive damage to the extracellular matrix [18]. In concert with this environmental deterioration, transcriptional analysis of isolated p16-expressing tissue-resident cells indicates up-regulation of pathways producing locally and systemically active soluble factors such as interleukins, chemokines, growth factors, and proteases [22,23,24,25]. Increased expression of these mediators in association with cell cycle arrest introduced the term “senescence-associated secretory phenotype” (SASP), to represent a prominent and proposed pathological feature of chronic cell senescence [3]. This senescence-associated reprogramming positions chronic senescent cells as accumulating sources of inflammatory and other soluble factors that alter microenvironments to exacerbate age-related deterioration within multiple tissues.

## 2. Inflammaging

Chronic inflammation, distinct from the transient inflammation that generally follows acute infection and insult, is the most common risk factor for mortality and morbidity in the elderly [26]. Low-grade, persistent inflammation often accompanies age-related disorders such as Alzheimer’s disease, atherosclerosis, diabetes, osteoporosis, cardiovascular disease and cancer [21,27,28,29,30,31,32]. Age-related diseases are not only exacerbated by inflammation but, in some cases, inflammation may actually be integral to their development. A causal relationship between inflammation and unhealthy aging was encapsulated under the term “inflammaging” [33]. Striking changes in levels of proteins involved in immune cell activation, recruitment and differentiation were detected together with various other age-associated changes in plasma composition in longitudinal human cohort studies of inflammaging. Modest increases in pro-inflammatory cytokines like tumor necrosis factor (TNF)-α, interleukin (IL)-6, and IL-1β consistently occur with age [4,19,34]. These elements of inflammaging are produced mostly by senescent endothelial cells somehow damaged or having proliferated to critical telomere lengths, now broadcasting distress signals to promote their clearance by immune cells. This chronic systemic inflammation that increases with age imposes persistent, pleiotropic and potentially deleterious effects on immune and other cells.

The cytokine milieu character within senescent tissue largely stems from the specific SASP manifested in resident cell types. As these inflammatory secretions are often a form of distress signaling, the increase in chronic inflammation that directly results from senescent cell accumulation is compounded by any age-related decline in immune function that compromises clearance of the accumulating pro-inflammatory cells [35]. This interrelationship between cellular senescence, chronic inflammation and immune function couples immunosenescence to accumulation of senescent cells and progressive, broadening degeneration of tissue function. Therefore, the senescence of immune cells themselves may be a critical benchmark in age-related physiological deterioration.

## 3. Immunosenescence

The term immunosenescence was originally coined in reference to elderly individuals’ weak responses to vaccines [36,37], but now is applied to multiple features of immunity affected by age and/or experience. This misnomer of sorts loosely describes the gradual decline in naïve T cells, increase in terminally differentiated T cells, reduction in T-cell repertoire diversity, rise in inflammation, fall in resistance to infection and deterioration in vaccine responsiveness [38]. Immunosenescence, as reflected by these systemic characteristics, encompasses macrophysiological changes in immune cell subset composition. More specifically, it is embodied in the accumulation of cells representative of terminal stages in adaptive immune cell memory. However, even when phenotypically alike, immune cells circumscribed within such a broad category as terminal memory often comprise a broadly heterogeneous population. Therefore, this conception of immunosenescence does not integrate senescence at the level of single cells, and these systemic changes do not necessarily reflect the same underlying relationship with cellular senescence as occurs in other tissues. Indices of cellular senescence, such as telomere attrition, as extrapolated from studies of fibroblast senescence, can be applied to immune cells to define a “single-cell theory” of senescence that underlies systemic functional and compositional changes within the aged immune system. In order to describe how adaptive immune cells progress towards senescence, we first discuss T-cell ontogeny and outline how their biology places them at unique risk for telomere-dependent chronic senescence.

Extensive proliferation of adaptive lymphocytes occurs during almost every stage of their development. Thymocyte progenitors emerge in the bone marrow and migrate to the thymus, still lacking antigen-specific T-cell receptors (TCR). Through staged processes of genetic rearrangement, proliferation and selection known as thymic selection, the TCRs generated ultimately determine the fate of thymocytes bearing them. For simplicity, only TCRαβ-expressing T cells are discussed in this review as their thymic selection is relatively well understood. Thymocytes rearrange individual germ-line gene segments within α and β loci to assemble composite TCR-encoding genes, with intense proliferation following successful rearrangement(s). Thymocytes at this stage express both CD4 and CD8 and undergo successive positive and negative selection to generate a final repertoire of T cells that can sensitively discriminate self-peptides from foreign peptides in the context of self-human histocompatibility-linked leukocyte antigen (HLA) molecules. As few as 2% of thymocytes survive this stringent multistage thymic selection process to enter the periphery, therefore, high levels of proliferation must occur at each stage to ensure sufficient numbers for the next round of selection and ultimate generation of a sufficient peripheral T cell repertoire [39]. Growth factors secreted by thymic stromal cells, such as IL-7, promote thymocyte proliferation and survival. Telomerase expression is enhanced by IL-7:IL-7R engagement to ensure these high levels of proliferation do not prematurely produce telomere erosion [40,41]. Only after highly stringent selection processes do immature thymocytes develop into mature, yet antigen naïve, T cells and enter the periphery.

In the periphery, naïve T cells constantly traffic throughout the blood and lymphatic system. A wide variety of chemokine and adhesion molecule receptors control their tissue homing and extravasation into lymph nodes. Signals needed for primary CD8^+^ T-cell activation are generated from TCR recognition of HLA class I molecule/peptide complexes and costimulatory interaction between T cell CD28 and CD80/86 on antigen-presenting cells (APC). The TCR complex is comprised of clonotypic α and β chains clustered with non-polymorphic CD3 proteins with intracellular immunoreceptor tyrosine-based activation motifs (ITAMs) capable of initiating signaling cascades. Activated kinases phosphorylate intracellular domains of proximal CD28 molecules to trigger interleukin-2 (IL-2) production and secretion, driving cell division and differentiation. Autocrine or paracrine engagement of IL-2 receptor (IL-2R) initiates telomerase expression and allows for rapid and extensive proliferation of activated T cells [42,43].

Effector CD8^+^ T cells primed in the lymph nodes survey peripheral tissue to identify host cells expressing cognate peptide/HLA class I complexes. Recognized peptides could be mutated self-proteins, potentially indicative of cancer, or viral proteins, reflecting viral replication within the host cell. Once these cells are identified, effector functions can be triggered, and target cells can be selectively removed. These effector functions utilize cytotoxic proteins such as granzymes and perforin, interferon (IFN)-γ and TNF-α and cell death receptors Fas/FasL to trigger pro-apoptotic caspase-mediated cell death pathways [44]. Pathogen clearance leads to contraction of the effector T cell population generated by reduced antigenic exposure and accessory signals. Regulatory contraction mechanisms depend on antigen withdrawal, falling cytokine and chemokine levels (in particular, IL-2, and other common γ chain cytokines) and on fratricide (T cell:T cell engagement through Fas:FasL interaction). These processes leave fewer than 5% of the effector CD8^+^ T cells generated in a primary response to survive as long-lived memory T cells [45]. Contraction of the proliferative effector pool into a small memory pool essentially recapitulates, in the periphery, the thymic selection events occurring in later stages of T-cell development.

Memory CD8^+^ T cells exhibit a phenotype and homing pattern distinct from naïve and effector T cells. Decreased CD62L and CCR7 (lymph node homing receptor) expression levels reflect the fact that these cells have undergone antigen priming and no longer require migration to peripheral lymph nodes for activation. Memory T cells also gain expression of CD27, which provides co-stimulatory signals upon antigen encounter in the periphery. Memory T cells retain expression of low affinity IL-2R (CD122) and IL-7R (CD127) chains, which allow for essential survival signals as well as periodic homeostatic and antigen-driven proliferation [41,46]. Intermittent proliferation occurs due to a variety of factors including (re-)infection, stress, lymphopenia or increased expression of immunostimulatory molecules. Memory T-cell persistence entails further phenotypic changes. Expression of CD28, CD27, CD122, and CD127 decreases in parallel with capacity to activate pro-survival mechanisms such as telomerase expression [47].

**Figure 2:**
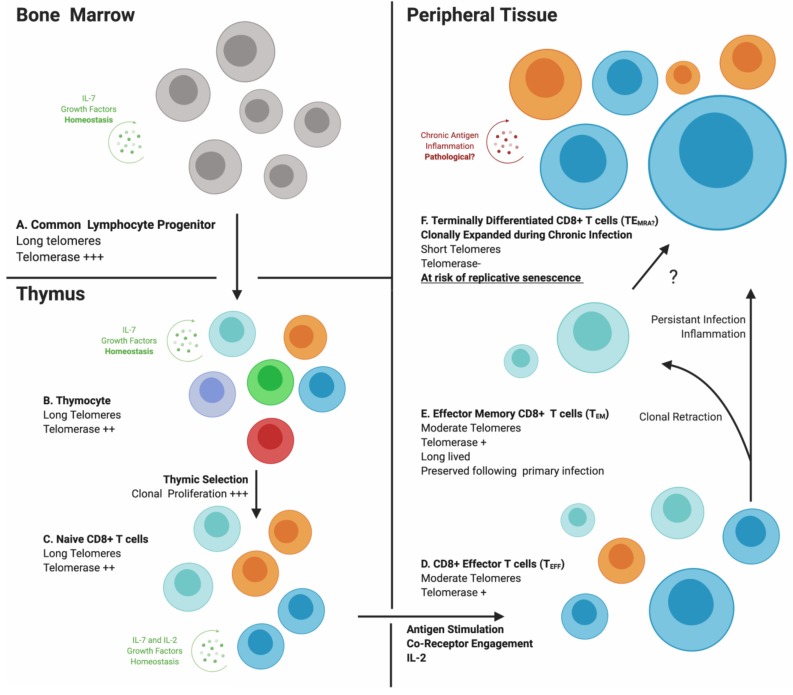
Factors involved in CD8^+^ T cell telomere maintenance. Lymphoid progenitor cells undergo cycles of quiescence and proliferation (**A**), the latter being maintained by high levels of telomerase expression initiated by cytokines and growth factors such as interleukin (IL)-7, secreted by bone marrow stromal cells. Similarly, thymic stromal cells supply IL-7, ensuring telomerase expression during the later proliferative stages of positive and negative selection (**B**), and in antigen-naïve CD8^+^ T cells (**C**). In the periphery, naïve CD8^+^ T cells encounter the antigen, with activation triggering a proliferative response via autocrine and paracrine IL-2:IL-2 receptor (R) interaction, and differentiate into CD8^+^ effector T (T_eff_) cells (**D**). IL-2R triggers telomerase expression in CD8^+^ effector memory T (T_em_) cells, allowing cellular preservation. However, continual activation reduces telomerase levels, leaving clonally expanded terminally differentiated CD8^+^ T cells (**E,639 F**) with undetectable levels of telomerase placing this subset at risk for critical telomere erosion and chronic senescence. (Created with BioRender).

From bone marrow through thymus to periphery, multiple regulatory processes affecting T lymphocyte proliferation, selection and survival work to ensure sufficiency of adaptive immune responses against pathogens. However, once antigen has been encountered and activation taken place, T cells begin a slow progression towards the replicative senescence illustrated by the in vitro fibroblast behavior described by Hayflick et al. [1]. With antigen-specific activation as the trigger, it is logical to envision proliferative phases of the adaptive immune response contributing to replicative senescence of individual immune cells and immunosenescence as a global characteristic reflecting the accumulation of senescent lymphocytes. As outlined in Figure 2, lymphocytes undergo intermittent periods of increased cell division between long quiescent phases, depending on antigenic exposure and levels of immunostimulatory molecules. Most lymphocytes (excluding NK cells) originate from single cells selected in the thymus or bone marrow based on the affinity and specificity of clonotypic receptors, and are selected on this basis again in the periphery during primary antigenic exposure (denoted in Figure 2 with a single color representing a single “clone”) and possibly during the effector cell contraction phase. Therefore, all T cells expressing the same antigen-specific receptor (TCR) theoretically are progeny of a single “clone.” This concept, termed clonal selection (denoted in Figure 2 by increased size of cells), specifies that each round of proliferation possibly contributes to telomere loss. During clonal expansion of naïve lymphocytes in primary and secondary lymphoid organs, telomerase activity is elevated through IL-7:IL-7R or IL-2:IL-2R engagement to compensate for telomere erosion during rapid cell division [41]. In the periphery, memory cells receiving antigenic stimulation also increase telomerase expression. However, this capacity is not conserved, but decreases with each successive round of proliferation [48] (Figure 2D–F). Telomerase expression decreases virtually to zero ex vivo in mitogen-activated memory T cells of the elderly, illustrating the age-related accumulation of a large subset of T cells at risk for harmful telomere erosion [49].

These observations support a direct mechanistic link between extensive immune cell proliferation, telomere erosion and in vivo immunosenescence. To investigate associations between immune cell telomere length and age-related illness, Cawthon et al. studied a large cohort of elderly patients stratified by whole-blood leukocyte telomere length over 15 years. Individuals with the shortest leukocyte telomere lengths had the lowest rate of survival and greatest risk of developing age-related morbidities, mostly involving cardiovascular or immune complications [50]. Determining the specific mechanism(s) linking telomere attrition to age-related functional T-cell decline could validate use of the term immune senescence and help clarify its role in inflammaging and age-related morbidity. Furthermore, identifying specific pathogens, other antigens or conditions that favor clonotypic expansion of memory T cell subsets towards senescence would be beneficial for developing strategies to delay or prevent immunosenescence.

## 4. Cytomegalovirus Infection Generates a Unique CD8^+^ T-Cell Subset

Human cytomegalovirus (CMV), a member of the beta subfamily of *Herpesviridae*, infects ~50% of the developed world’s population. Large and robust immune responses incorporating natural killer (NK) cells, inflammatory cytokines, B cells and T cells contribute to virus control after primary infection, but CMV persists by establishing latent infection. Memory CD8^+^ T cell responses to CMV have been extensively studied, largely due to their atypical expansion, termed memory inflation, into as much as 90% of total memory CD8^+^ T cells [51]. Memory inflation also occurs in CMV-specific CD4^+^ T cell populations, but to a much lesser extent [52]. Although these populations are thought to relate to lifelong infection and periodic CMV reactivation from latency, the mechanisms behind these expansions and how they are maintained are poorly understood. As CMV-specific CD8^+^ T cells undergo massive clonal proliferation, display an altered phenotype and are functionally abnormal, we suggest that cells within this population may illustrate the transition to in vivo replication driven senescence in T lymphocytes.

Long-term cultured CD8^+^ T cells lose CD27 and CD28 expression [53] and, thus, it is assumed that CD8^+^ T cells lacking expression of either in vivo are long-lived memory cells. The CMV-specific memory T cells are primarily CD28^-^CD27^-^CD8^+^ [54] with no dependence on costimulatory signals for activation [55]. Ex vivo stimulation of CD28^-^CD8^+^ T cells can restore expression of CD28, possibly to help initiate paracrine IL-2 activity. However, this does not occur for antigen-stimulated CD28^-^ CMV-specific T cells, as transduction with a CD28 expression vector was required for IL-2 production and proliferation [56]. Increased CD57 expression is strongly associated with loss of CD27/28 [54] and also with a loss of IL-2 family receptors CD122 and CD127 [44,57,58], all predisposing towards lesser proliferative capacity and telomerase expression. Most CMV-specific CD8^+^ T cells express CD57 [59], a cell surface glycoprotein associated with extensive proliferation and what is referred to as “terminal differentiation.” On NK cells, CD57 expression identifies populations with greater natural and antibody-dependent cytotoxicity responses, which undergo lesser proliferation when exposed to activating cytokines such as IL-12 or IL-18 [60]. Increased expression of CD57 and decreased CD28, IL-7R, and IL-2R expression signify that CMV-specific CD8^+^ T cells have undergone extensive proliferation and lack the signaling molecule required for telomerase induction. Thus, conditions under which pre-senescent or senescent T cells with eroded telomeres develop are operative in this population. However, this perspective is complicated by demonstration that CMV-specific CD8^+^ T cells expressing lacking CD28 and expressing CD57 proliferate well when alternative costimulation is provided by 4-1BB [61]. In fact, most cases of poor proliferation observed ex vivo with antigen-specific T cells are not directly due to the lack of telomerase or telomere shortening, but are imposed by expression of inhibitory receptors or requirements for alternative forms of stimulation [61,62,63]. Even T cells expressing the “terminal differentiation” marker CD57, including CMV-specific CD8^+^CD28^-^ cells, proliferate well in vitro with appropriate stimulation [61]. Thus, the dysfunction reported for antigen-specific effector memory T cells ex vivo is not attributable to cellular senescence per se, but to shifting requirements for costimulation that accompany successive cycles of proliferation. This memory progression features reduced capacity for telomerase expression and induction of inhibitory receptors that attenuate activation and contribute to the general features of immunosenesence associated with chronic infection/antigenic stimulation (Figure 2E,F). Telomere shortening does occur in parallel and presumably limits the proliferative potential of CD28^-^CD57^+^ antigen-specific T cells.

The secretory profile and antiviral responses of CD8^+^ T cells are directly influenced by their stage of differentiation and nature of the viral interaction. As CMV-specific CD8^+^ T-cell frequency rises over time, there is speculation that recurrent subclinical rounds of antigenic clearance reinforce the multifunctionality of CMV-specific CD8^+^ T cells optimized for effector function [64,65]. While capable of robust cytokine production and cytotoxicity, they also exhibit a poor proliferative response [62,63,66]. Antigen-exposed CMV-specific CD8^+^ T cells simultaneously produce IFN-γ and TNF-α, faster and at higher levels than other virus-specific CD8^+^ T cells [66,67]. In addition, CMV-specific T cells demonstrate higher immediate production of granzyme/perforin and higher levels of pre-assembled granules, reflecting their capacity to exert prompt, robust cytotoxic activity against CMV-infected cells [58,66,68]. During chronic human immunodeficiency virus (HIV) and/or hepatitis C virus (HCV) infection, a majority of CD8^+^ T cells recognizing HIV or HCV associated epitopes produce antiviral cytokines such as IFN-γ, but display a reduced ability to lyse viral peptide-pulsed target cells, while those directed against CMV retain robust cytotoxicity in the same host [69]. Selective maintenance of CMV-specific T cells with these features could represent adaptation towards greater viral control or acquisition of secretory features (SASP) representative of replicative T-cell senescence.

Their abundance and other features of CMV-specific CD8^+^ T cells favor their utility in exploring and understanding in vivo progression to replicative T-cell senescence. This effector memory subset exhibits atypical functional specialization, potentially embodying features of an adaptive immune cell SASP. To reach their level of abundance, CMV-specific T cells undergo extensive in vivo proliferation and then display certain defects in ex vivo replication, potentially foreshadowing cellular senescence due to telomere shortening. Expression of similar traits to those observed in senescent fibroblasts by Hayflick et al. suggests that CMV-specific T cells could be a peri-senescent immune cell subset. A study of lymphocyte telomere length in HIV-infected individuals positioned CMV-specific CD8^+^ T cells as having the shortest telomeres and highest rate of telomere attrition [70]. Therefore, the accumulation of such immune cells through extensive proliferation could underlie a relationship between CMV infection and age-associated morbidity.

## 5. CMV-Specific CD8^+^ T Cells: The Link Between CMV Infection and Age-Related Morbidity

In a representative population of the developed world (*n* = 21,639), the prevalence of CMV infection ranged from 36% in children under 11 to 91% in adults over the age of 80 [71]. In all age groups, CMV infection can trigger memory inflation of the adaptive immune response and in the old elderly age group, this memory inflation conveys a greater risk for all-cause mortality [72]. It has been suggested that through its progressive impact on the T-cell repertoire, CMV infection accelerates aging of the immune system, which accelerates unhealthy aging in general. We now focus on the relationship between CMV infection and previously described features associated with immunosenescence, including phenotypic changes and reduced diversity in the T-cell repertoire, increased inflammation, and increased risk for multiple age-related morbidities.

Thymic involution reflects structural and functional degeneration of the thymus that begins around puberty and continues into old age. Thymic tissue is gradually replaced by adipose tissue, reducing output and largely restricting naïve T-cell production to prepubescence. This age-associated decline in thymopoiesis reduces the ratio of naïve to memory T cells and in concert with antigenic experience, produces the changes in immune profile that occur with age [73] and characterize immunosenescence. Given its prolific activity early in life, older children and adults are generally considered to be unaffected by thymic removal. However, a study by Sauce et al. showed that this is not true for thymectomized children infected with CMV [74]. Thymectomized CMV-seropositive children had an accelerated fall in naïve:memory T-cell ratios to levels normally seen in old age. Deep sequence profiling the TCR repertoires of these children revealed relative TCR restriction, with a majority of sequences complementing immunodominant CMV antigens, again replicating a feature associated with immunosenescence in the elderly [75]. These results reflect CMV infection prematurely inducing immune alterations commonly associated with aging. Natural thymic decline is also accelerated by CMV infection, as TCR diversity analyses of healthy CMV-seropositive adults revealed 33% reductions in TCR diversity compared to seronegative individuals of the same age [59]. A more recent study of TCR sequences in the elderly demonstrated similar dominance of T cell clones specific for CMV, but also showed maintenance of low frequency clones in the T cell repertoire [76]. Thus, loss of diversity in the T cell repertoire may not be absolute, despite overwhelming dominance of a few CMV-specific clones. These studies indicate that CMV infection or, more specifically, the immune response to CMV infection, can accelerate the onset of age-related relative reductions in TCR diversity associated with immunosenescence.

High frequencies of peri-senescent CMV-specific CD8^+^ T cells constitute a potential source of inflammatory mediators that contribute to inflammaging. “Healthy” CMV seropositive individuals tend to have low-grade chronic inflammation characterized by subtle increases in the levels of T helper 1 (TH1) type cytokines, growth factors, IL-6, and C reactive protein (CRP) [77]. Sustained increases in CRP, an acute phase protein normally produced by the liver during acute infection in response to IL-6, IL-1β or TNF-α, indicate increased risk for cardiovascular disease [78]. A study by van de Berg et al. associated serum levels of IFN-γ with CRP during CMV infection, supporting suggestions that the magnitude of CMV-specific T cell immunity is associated with increased risk for cardiovascular disease [77]. Not all studies have found a relationship between CMV infection and inflammation or mortality in the aging population [34]. The BELFRAIL study of old elderly found no influence of CMV infection on mortality in the old elderly population studied [79]. While this could represent a selection effect, where susceptible individuals did not reach the age of entry to the study or the fact that only seropositivity for CMV was considered, rather than the nature of the T cell response against CMV. In the Swedish octogenarian (OCTO) and nonagenerian (NONA) longitudinal population aging studies, Pawelec et al. directly associated changes in immune parameters caused by CMV infection with increased mortality in the elderly, alluding to the role of immunosenescence. These studies established relationships of immune parameters such as an inverted CD4^+^/CD8^+^ T-cell ratio, decreased T-cell repertoire diversity and increased systemic inflammation with mortality [37,59,68]. More specifically, where cause of death was available, these studies drew attention to dramatic increases in inflammation and cardiovascular disease-associated deaths in a subset of CMV-infected elderly [80,81]. Infection with CMV is linked to increased risk of multiple age-associated morbidities in the general population, especially diabetes, atherosclerosis, and cardiovascular disease [82]. Independent of age, infection with CMV is associated with a 4-fold increase in new diabetes cases in kidney-transplant recipients, a risk not seen with any other viral infection [83]. Diabetes is compounded by the increased inflammation that accompanies CMV infection, raising the risk for atherosclerotic events with clinical repercussions [30,84]. A study exploring the additive potential of diabetes and CMV infection with regard to atherosclerosis, found that anti-CMV IgG titers paralleled the frequency and severity of cardiovascular events [85]. Therefore, systemic inflammation occurring during CMV infection may exacerbate autoimmune and other chronic inflammatory conditions. Since CMV-specific T-cell immunity increases with age through the process of memory inflation, the immune response against CMV may be the key factor influencing aging of the immune system and the downstream consequences. Vascular degeneration due to circulating peri-senescent, pro-inflammatory CD8^+^ T cells could underlie the link between CMV infection and increased age-associated risk for cardiovascular disease. Despite extensive literature on how CMV infection affects the adaptive immune system, the mechanistic connection to increased risk for multiple age-related morbidities remains obscure.

The profound influence that CMV infection can have on the immune system is not uniformly negative. Its ability to stimulate exceptionally strong CD8^+^ T cell effector memory responses has been employed in an especially promising HIV vaccine candidate [86,87]. Mice latently infected with murine (M) CMV had enhanced protection against bacterial infections, presumably mediated by an increased background of innate immune activation [88]. Although somewhat controversial, a number of human clinical studies have demonstrated an association between HCMV reactivation following bone marrow transplantation and reduced relapse rates of leukemias and lymphomas [89,90,91]. Multiple mechanisms are thought to be at play in these cases related to the known effects of CMV on activation of NK cells [92], CD8^+^ T cells and γδ T cells [89] as well as the potential for both direct and immunostimulatory oncolytic effects [93].

## 6. HIV-Associated “Premature” Aging and CMV Co-Infection

Persons living with HIV (PLWH) are commonly referenced as suffering premature immunosenescence [94]. Guaraldi et al. reported that individuals with chronic HIV infection often suffer from two or more additional age-associated morbidities compared to age-matched uninfected individual [17,61]. Chronic inflammation and immune activation have been suggested as causes of premature aging in HIV infected individuals. While there are a number of candidate factors for increasing inflammation in chronic HIV infection, the prevalence of CMV infection and its exaggerated effects in this setting point to evaluation of the relationship between CMV infection, immunity against CMV and accelerated aging.

Early studies of CMV infection in PLWH in the pre-highly active antiretroviral therapy (HAART) era identified it as a potential cofactor accelerating progression to acquired immune deficiency syndrome (AIDS) and its symptomatic reactivation was categorized as an AIDS-defining illness [95]. This role of CMV as an opportunistic infection in the context of HIV infection differs in nature from the proposed deleterious age-associated effects CMV has on inflammation and the immune system of elderly HIV-negative individuals. Therefore, the potential contribution of CMV infection and the proliferative burden it imposes on the adaptive immune system to “premature” aging in HIV infection are assessed in a different light than as an opportunistic infection. A recent study found coinfection with CMV was clearly associated with immunosenescence, but not with inflammation in PLWH [96]. Most studies have found an association between coinfection with CMV and increased inflammation [70,97,98,99] and a large study reported increased risk for non-AIDS related clinical events with CMV coinfection [100].

There are many parallels between the immunological effects of HIV infection and CMV infection. As in the CMV-infected elderly with memory inflation, HIV infection is associated with increased absolute numbers of memory CD8^+^ T cells [101,102]. This persistent increase in memory CD8^+^ T cells occurs despite effective treatment that reduces HIV replication and permits restoration of CD4^+^ T-cell counts [102]. Also like the CMV-infected elderly, HIV-infected individuals have decreased relative T cell receptor TCR repertoire diversity compared to the general population, particularly in the CD8^+^ T cell memory compartment [103]. Together, these features suggest extensive clonal expansion or selective homeostatic proliferation such as occurs with CMV infection, but over a much shorter timeframe. The CD8^+^ T-cell population in HIV-infected individuals shows broad increases in CD57 expression, decreases in co-receptor expression (CD27, CD28) and a reduced capacity to proliferate and mount effector functions ex vivo compared to CD8^+^ T cells from uninfected individuals [54,104]. Although similar to the immunological abnormalities associated with the extensive clonal expansion of CMV-specific T cells in elderly non-HIV-infected individuals, mechanistic connections between these phenotypes have not been extensively examined. It is common to use the ratio of CD4^+^/CD8^+^ T cells as a surrogate marker for immune competence in HIV infection and when this ratio falls or remains below 1.0, there is a higher risk for non-AIDS related adverse clinical events [105]. Early-exposed HIV-infected children co-infected with CMV have higher frequencies of terminally differentiated CD8^+^ T cells and lower CD4^+^/CD8^+^ T-cell ratios than CMV-seronegative children, regardless of detectable CMV DNA or HIV RNA in their peripheral blood [92]. This observation indicates that at least under some circumstances, CMV infection contributes to an age-independent expansion of CD8^+^ T cells in HIV infection. Barrett et al. further illustrated the impact of CMV infection in PLWH, showing that CMV-seropositive PLWH have lower CD4^+^/CD8^+^ T-cell ratios, higher frequencies of CD57^+^CD8^+^ T cells and lower frequencies of CD28^+^CD8^+^ T cells, independent of age and HIV viral load [102]. Co-infection with CMV clearly increases characteristics of senescence within the CD8^+^ T cell repertoire of PLWH in an age-independent manner.

Leukocyte telomere length has been investigated in relation to the premature aging associated with HIV infection. Telomere erosion is markedly increased in HIV infection, even when compared to that of uninfected individuals who are decades older. By compartment, CD8^+^ T cells possess shorter telomeres [106] and have fewer T cell receptor excision circles [107] than CD4^+^ T cells, indicating greater overall proliferation of CD8^+^ T cells in HIV infection. Effector memory CD8^+^ T cells that re-express CD45RA or CD8^+^ T_EMRA_, a subset comprised of pro-inflammatory terminally differentiated effector T cells, have shorter telomeres than other memory subsets, as do CD57^+^CD8^+^ T cells [108]. Although CMV infection is associated with increased numbers of circulating CD8^+^ T_EMRA_, it was not taken into consideration for these studies. However, a subsequent study identified CMV-specific CD8^+^ T cells as having the shortest telomeres and undergoing the most rapid telomere erosion in PLWH [70]. Leukocyte telomere length is also associated with duration of untreated HIV disease and lower CD4^+^ T cell nadirs [109]. As these same factors are associated with decreased TCR diversity, this suggests a relationship between lymphocyte telomere length and TCR diversity, potentially effected through CMV-related T-cell clonal expansion. Furthermore, leukocyte telomere length in PLWH is inversely associated with risk of heart disease, type 2 diabetes, cancer, and a host of other age-associated morbidities [110], reflecting the similar relationship as occurs in the older CMV-infected, HIV-negative population. These associations implicate CMV-related clonal expansion as a primary driver of lymphocyte telomere erosion affecting the earlier and increased risk for age-related diseases in HIV infection. Accelerated telomere loss directs the focus onto exhaustive proliferation of CD8^+^ T cells directed against CMV epitopes as an integral factor contributing to senescence within the CD8^+^ T-cell population in HIV infection.

## 7. Conclusions

Clearance of senescent cells is an important role of the adaptive immune system in relation to healthy aging. This regulatory role can be compromised by aging itself, HIV infection or many other immune stressors that weaken immune function and/or promote immunosenescence. The deficits in adaptive immunity observed in HIV/CMV co-infection, accompanied by an increased prevalence of age-associated pathologies termed accelerated aging, together comprise a unique setting within which to study senescent cell accumulation and its physiological impact. Immunological stress, as imposed by chronic infection, chronic inflammation or requirements for homeostatic proliferation, can promote progression of lymphocytes to cellular senescence and impose immune deficits that impair clearance of senescent cells from tissues, thus, compounding the accumulation of senescent cells and the negative implications of their SASP. The homing of abundant circulating CMV-specific CD8^+^ T cells to such inflamed tissues could fuel a further feedback loop of activation, proliferation and telomere attrition. Therefore, not only are dysfunctional immune cells indirectly contributing to senescent cell accumulation, but are themselves progressing towards senescence, reinforcing a vicious cycle that may be a key factor in “premature aging” of PLWH and unhealthy aging in others. If CMV infection is a major factor in shifting the adaptive immune system towards chronic senescence, the most dramatic effects should be revealed in the context of HIV infection, where increased chronic inflammation and exaggerated anti-CMV immune responses may optimally promote progression of age-independent chronic immunosenescence. Within this setting, CMV infection is associated with CD8^+^ T-cell progression towards cellular senescence, increased inflammation and greater risk of age-related morbidities. This may represent acceleration of the same effects that CMV infection produces in old elderly individuals who develop an immune risk profile (IRP) with its connotations for immunosenescence, unhealthy aging and mortality. Extensive proliferation and oligoclonal dominance of CMV-specific CD8^+^ T cells are the hallmarks and potentially drivers of these associations.
